# Association between prehypertension, metabolic and inflammatory markers, decreased adiponectin and enhanced insulinemia in obese subjects

**DOI:** 10.1186/1743-7075-11-25

**Published:** 2014-06-02

**Authors:** Amanda Roberta de Almeida, Sarah Monte-Alegre, Michele Bianca Zanini, Aglécio Luiz Souza, Maurício Etchebehere, José Antonio Rocha Gontijo

**Affiliations:** 1 Disciplina de Medicina Interna, Laboratório de Metabolismo Hidrossalino, Universidade Estadual de Campinas, 13083-592 Campinas, SP, Brasil; 2 Departamento de Clínica Médica, Faculdade de Ciências Médicas, Universidade Estadual de Campinas, 13083-592 Campinas, SP, Brasil

**Keywords:** Obesity, Metabolic syndrome, Blood pressure, Inflammation, Adiponectin, Renal function

## Abstract

**Background:**

Obesity is associated with development of the cardiorenal metabolic syndrome, which is a constellation of risk factors, such as insulin resistance, inflammatory response, dyslipidemia, and high blood pressure that predispose affected individuals to well-characterized medical conditions such as diabetes, cardiovascular and kidney chronic disease. The study was designed to establish relationship between metabolic and inflammatory disorder, renal sodium retention and enhanced blood pressure in a group of obese subjects compared with age-matched, lean volunteers.

**Methods:**

The study was performed after 14 h overnight fast after and before OGTT in 13 lean (BMI 22.92 ± 2.03 kg/m^2^) and, 27 obese (BMI 36.15 ± 3.84 kg/m^2^) volunteers. Assessment of HOMA-IR and QUICKI index were calculated and circulating concentrations of TNF-α, IL-6 and C-reactive protein, measured by immunoassay.

**Results:**

The study shows that a hyperinsulinemic (HI: 10.85 ± 4.09 μg/ml) subgroup of well-characterized metabolic syndrome bearers-obese subjects show higher glycemic and elevated blood pressure levels when compared to lean and normoinsulinemic (NI: 5.51 ± 1.18 μg/ml, P < 0.027) subjects. Here, the combination of hyperinsulinemia, higher HOMA-IR (HI: 2.19 ± 0.70 (n = 12) *vs.* LS: 0.83 ± 0.23 (n = 12) and NI: 0.98 ± 0.22 (n = 15), P < 0.0001) associated with lower QUICKI in HI obese when compared with LS and NI volunteers (P < 0.0001), suggests the occurrence of insulin resistance and a defect in insulin-stimulated peripheral action. Otherwise, the adiponectin measured in basal period was significantly enhanced in NI subjects when compared to HI groups (P < 0.04). The report also showed a similar insulin-mediated reduction of post-proximal urinary sodium excretion in lean (LS: 9.41 ± 0.68% *vs.* 6.38 ± 0.92%, P = 0.086), and normoinsulinemic (NI: 8.41 ± 0.72% *vs.* 5.66 ± 0.53%, P = 0.0025) and hyperinsulinemic obese subjects (HI: 8.82 ± 0.98% *vs.* 6.32 ± 0.67%, P = 0.0264), after oral glucose load, despite elevated insulinemic levels in hyperinsulinemic obeses.

**Conclusion:**

In conclusion, this study highlights the importance of adiponectin levels and dysfunctional inflammatory modulation associated with hyperinsulinemia and peripheral insulin resistance, high blood pressure, and renal dysfunction in a particular subgroup of obeses.

## Background

Obesity, a public health problem of the first order for industrialized and non-industrialized countries dramatically cause a reduction in overall life expectancy [1]. The prevalence of obesity in different populations is related to environmental factors, such as reduced physical activity and diet, mainly. Nowadays, with growing industrialization and modern lifestyle, the access to high fat and carbohydrate diets has changed the eating habits of the population. These aspects interact with genetic factors, which may explain the excessive body fat throughout the world [2]. Overweight and obesity are related to increased prevalence of cardiovascular disease, dyslipidemia, type 2 diabetes mellitus, neurological disease, chronic kidney disease and cancers [3-5]. Obesity appears to be an important factor in elevating blood pressure in many prehypertensive individuals since studies have shown that high blood pressure is more prevalent in obese than in non-obese subjects [2-4]. In this way, experimental studies have demonstrated that weight gain, even over a period of a few weeks, consistently elevates blood pressure and weight loss decreases blood pressure independent of changes in sodium intake. Recently, study in our laboratory has demonstrated that podocyte injury in parallel with proteinuria and evidence of epidermal mesenchymal transition transformation are associated with long-term loss of kidney function and renal sodium and water retention in obesity induced by high-fat diet intake in rats [5]. However, establishing cause-and-effect relations has been hampered by the lack of suitable animal models that mimic obesity induced hypertension in humans and that allow sequential changes in renal, endocrine, and cardiovascular function to be monitored during the development of obesity. Although this association between obesity and hypertension is widely recognized, the mechanisms responsible for weight-related changes in blood pressure have not been elucidated. Also, sparse reports that deal with the relationship between inflammatory response, plasma insulin and cytokines levels and blood pressure in obese subjects are known to revision see ref. [6,7]. Additionally, overweight and obesity are associated with development of the cardiorenal metabolic syndrome which is a constellation of risk factors, such as insulin resistance, dyslipidemia, and high blood pressure that predispose affected individuals to well-characterized medical conditions such as diabetes, cardiovascular and kidney chronic disease [7-14]. However, the pathophysiological mechanisms and humoral factors involved in obesity and that lead to sodium retention and higher blood pressure in obeses are still unclear. Therefore, the current study was designed to establish relationship between metabolic and inflammatory disorder, renal sodium handling disorder and enhanced blood pressure in a group of obese subjects compared with age-matched, lean volunteers. To address the different parameters among lean and obese groups, the study was performed by examining oral glucose tolerance test, HOMA-IR and QUICKI index, renal tubule sodium handling and plasmatic inflammatory cytokine markers.

## Subjects and methods

### Subjects

Forty normotensive volunteers aged 18–50 years were enrolled in the study and divided into two subject groups: (1) 13 lean (LS, BMI 22.92 ± 2.03) and, (2) 27 outpatient obese (OS, BMI 36.15 ± 3.84) volunteers. Body mass index (BMI) was calculated based on ratio between body mass (in kg) and squared height (in meters) [weight (kg)/height (m^2^)]. On the basis of fasting plasma insulin and glucose levels homeostasis model assessment of insulin resistance (HOMA-IR), two distinct obese subgroup profiles were identified by HOMA-IR *z*-score, and subdivided in (3) 15 normoinsulinemic obese (NI, BMI 35.25 ± 3.80 kg/m^2^) and (4) 12 hyperinsulinemic obese (HI, BMI 37.05 ± 3.88 kg/m^2^). The present study evaluated the body composition measurements using bioelectrical impedance analysis and the energy expenditure was assessed by continuous indirect calorimetry performed with a calorimeter (SensorMedics Corp., Anaheim, California, USA). The characteristics of these experimental groups are presented in Table 1. A complete medical examination was carried out to exclude arterial hypertension (their office systolic and diastolic pressure should be lower than 140 and 90 mmHg, respectively, when measured on at least three occasions) and personal history of diabetes mellitus (fasting glucose less than 5 mM), or chronic renal, liver and others endocrine diseases. Both lean and obese subjects had negative first degree familiar histories of diabetes mellitus and arterial hypertension. None of the volunteers had shown recent changes in the body mass or dietary habits, and they were asked to continue their normal activities throughout the study. The female participants were studied during the follicular phase of the menstrual cycle. Other exclusion’s criteria for all the subjects were: steroidal and non steroidal anti-inflammatory therapies, hormonal substitutive or contraceptive therapy, hormonal therapy for any thyroid dysfunctions, drugs or alcohol abuse, smoking and mental disability. The protocol *(#1221/2009)* was approved by Institutional Review and Ethics Committee of Medical Science School at Campinas State University and all subjects gave their consent to participate of the study, which was conducted according to the guidelines for human experimentation established by the Declaration of Helsinki (2013) and Brazilian laws on this matter.

**Table 1:** Subject’s general clinical characteristics for obese and lean groups

	**Control**	**n**	**Normoinsulinemic**	**n**	**Hyperinsulinemic**	**n**	**p**	**p**^**1**^	**p**^**2**^	**p**^**3**^
	**(LS)**		**(NI)**		**(HI)**			**LS** ***vs.*** **NI**	**LS** ***vs.*** **HI**	**NI** ***vs.*** **HI**
M/F*	4/9	13	2/13	15	5/7	12	ns	-	-	-
Age (years)	33 ± 12	13	29 ± 7	15	29 ± 8	12	ns	ns	ns	ns
Body mass (Kg)	63.71 ± 8.98	13	95.67 ± 12.78	15	103.464 ± 12.89	12	0.0001	0.0001	0.0001	ns
Height (m)	1.66 ± 0.10	13	1.63 ± 0.09	15	1.69 ± 0.09	12	ns	ns	ns	ns
BMI (kg/m^2^)	22.92 ± 2.03	13	35.25 ± 3.80	15	37.05 ± 3.88	12	0.0001	0.000	0.0001	ns
Fat mass (kg)	18.66 ± 3.98	13	36.70 ± 7.15	15	40.80 ± 9.24	12	0.0001	0.0001	0.0001	ns
Lean mass (kg)	41.49 ± 6.02	13	57.22 ± 8.02	15	63.76 ± 10.12	12	0.0001	0.0001	0.0001	ns
Body water (%)	48.77 ± 4.98	13	41.34 ± 5.16	15	42.04 ± 4.72	12	0.006	0.004	0.008	ns
Basal metabolic rate (kcal)	1261.3 ± 183.53	13	1740 ± 244.07	15	1938.45 ± 307.62	12	0.0001	0.000	0.0001	ns

*M/F* male/female; *BMI* body mass index; Values are means ± DP; P values for comparison between groups by non-parametric analysis of Kruskal *Wallis*; *P values for comparison between groups by non-parametric analysis descriptive of *Chi-Square;* P, comparing all groups. P^1^, P^2^ and P^3^ comparison between groups.

### Experimental design and methods

The studies were performed after 14 h overnight fast during which water ingestion was permitted [8-10,15-17]. A single dose of 300 mg lithium carbonate was administered to all subjects 14 hours before the renal tests. At 8:00 a.m., each subject was asked to empty his bladder and to discard the urine. Immediately thereafter, diuresis was induced by an oral loading equivalent to 20 ml of tap water/kg body lean mass given between 8:00 and 8:45 h a.m. The subjects rested comfortably in the sitting position throughout the study and stood only to urine avoid. The urinary volume losses were replaced with drinking tap water every hour from 9:00 to 11:00 h a.m. Oral glucose tolerance test (*OGTT*) consisting of a 75 g glucose load as a 22% solution in water was performed concomitantly with renal tests in both lean and obese subjects. Venous blood was sampled at 0, 30, 45, 60, 90 and 120 minutes for determination of glucose and insulin plasma levels. For each subject, the following parameters were determined: blood pressure measured by a appropriated mercury cuff, using a stethoscope placed over the brachial artery for auscultation of pulse, according to the Task Force Report on High Blood Pressure (NHBPEP, 1996), BMI percentile, as well as serum concentrations of glucose, insulin, cholesterol total, triacylglycerol, high-density (HDL-cholesterol) low-density (LDL-cholesterol) and very low-density (VLDL-cholesterol) lipoproteins cholesterol. On the basis of fasting plasma insulin and glucose levels homeostasis model, assessment of insulin resistance (HOMA-IR) [11,18] and quantitative insulin-sensitivity check (QUICKI) [12,19] indexes were calculated. HOMA-IR and QUICKI were calculated according to the formulae: HOMA-IR = fasting insulin (μU/ml) × fasting blood glucose (mM)/22.5 and, QUICKI = 1/[log fasting insulin (μU/ml) + log fasting blood glucose (mg/100 ml)], i.e. 1/[log fasting insulin (μU/ml) + log fasting blood glucose (mM) × 18.182].

### Creatinine and lithium clearance

The creatinine clearance (*CCr)* used to estimate the glomerular filtration rate and the lithium clearance (*CLi*) used to estimate the sodium output from the proximal tubule were calculated by standard formulas (U.V)/P) where, U is the urinary creatinine and lithium concentrations, V is the urinary flow and P is the creatinine and lithium plasma levels. Fractional sodium (FE_Na_) and potassium (FE_K_) excretion were calculated as *C*_Na_/*C*_Cr_ × 100 and *CE*_*K*_*/CF*_*K*_ × 100, respectively, where *C*_Na_ is sodium clearance, *CE*_K_ is potassium clearance, CCr is creatinine clearance and CF_K_ is filtered load potassium. The fractional proximal (FEP_Na_) and post-proximal (FEPP_Na_) sodium excretion were calculated as *C*_Li_/*C*_Cr_ *×* 100 and *C*_Na_*/C*_Li_ × 100, respectively [5,7,13,14,20-22].

### Biochemical analysis

Plasma and urine sodium, potassium and lithium concentrations were measured by flame photometry (Micronal, B262, São Paulo, Brazil), while creatinine concentrations were determined spectrophotometrically (Instruments Laboratory, Genesys V, USA). The Glucose Analyzer YSI 2300 measured plasma glucose. Plasma concentrations of insulin and C-peptide were measured by immunoassay (Millipore, Billerica, USA, with sensibility of 1μU/ml; and 0,05 ng/ml, respectively). The HDL and LDL-cholesterol and, Triglyceride levels were also measured using enzyme immunoassay kits with a Modular Analytic P Biochemistry Analyzer (Roche®) according to the manufacturers' protocols. The cytokines were measured by immunoassay (adiponectin, Millipore, Billerica, USA, sensibility 0,155 ng/ml; IL-6 R&D SYSTEMS®, Minneapolis, USA, sensibility 0,039 pg/ml; leptin Millipore, Billerica, USA, sensibility 0,195 ng/ml; TNF-α R&D SYSTEMS®, Minneapolis, USA; CRP DIAsource ImmunoAssays S.A, Louvain-la-Neuv, Belgium, sensibility 10 ng/ml).

### Data presentation and statistics

All analyses were performed using SPSS (SPSS version 17.0). Data obtained from this study are expressed as the mean ± SD or median and quartile deviation when appropriated. The diagnostic checking was performed by analysis of the correlations of residues between the model and the samples. The integrated glucose and insulin secretion, i.e., the total area under curve (tAUC, mg/ml/120 min and μU/ml/120 min, respectively) was calculated by the trapezoidal method and we have used it to establish the statistical difference between the lean and subgroups of obese subject. Statistical analyses were performed using non-parametric analysis by Kruskal-Wallis test or Student *t*-test when appropriated from GraphPad Prism 5.01 (GraphPad Software, Inc., USA). Comparisons involving only two means within or between groups were carried out using a Student *t*-test. We also analyzed independent factors related to systolic blood pressure, body constitution and HOMA-IR index values with multiple linear regression analysis. The level of significance was set at *P* ≤ 0.05.

## Results

### Subjects characteristics

**Figure 1:**
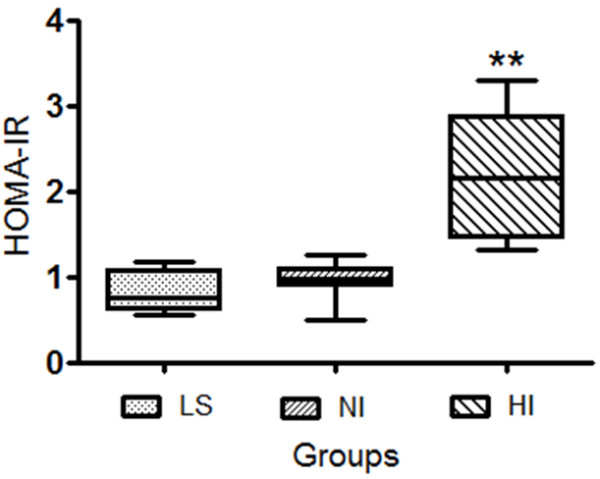
**Representation of HOMA-IR in control (LS, n = 12) and obese normoinsulinemic (NI, n = 15) and hyperinsulinemic (HI, n = 12) subgroups.** Data are expressed as median and quartile deviation. The level of significance was set at **P ≤ 0.001 *versus* LS and NI (non-parametric analysis by Kruskal-Wallis test).

**Figure 2:**
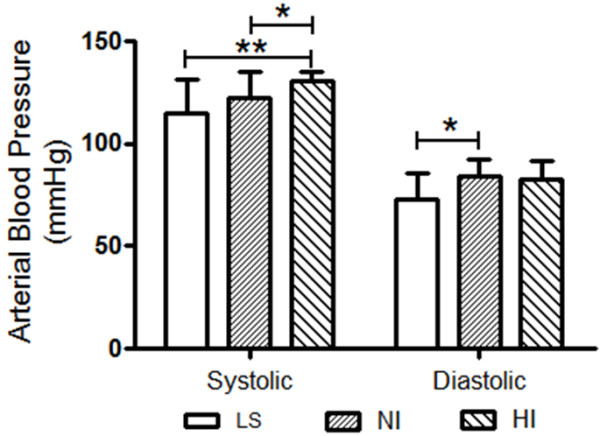
**Graphic representation of arterial systolic and diastolic blood pressure in control lean (LS, n = 13) subjects compared to normoinsulinemic (NI, n = 15) and hyperinsulinemic (HI, n = 10) volunteer subgroups.** Results are expressed as mean ± SD. The level of significance was set at *P ≤ 0.05 or **P ≤ 0.01 (non-parametric analysis by Kruskal-Wallis test).

The characteristics of the all groups included in this study are presented in Table 1. The three groups were well matched for age. The mean age of the lean and obese subjects was respectively, 33 ± 12 and 29 ± 7.5 years (NI, 29 ± 7.0; HI, 29 ± 8.0). Considering the gender distribution, 44.4% of LS and 35% of OS were male. On the basis of fasting plasma insulin and glucose levels homeostasis model assessment of insulin resistance (HOMA-IR), two distinct obese subgroup profiles were identified by HOMA-IR z-score, and subdivided in normoinsulinemic obese and hyperinsulinemic obese volunteers (Figure 1). The mean HOMA-IR index value were significantly higher in HI obese subgroup when compared to LS and NI [HI: 2.19 ± 0.70 (n = 12) *vs.* LS: 0.83 ± 0.23 (n = 12) and NI: 0.98 ± 0.22 (n = 15), P < 0.0001]. The BMI differed significantly (P < 0.0001) between LS (22.92 ± 2.03) and both normoinsulinemic (35.25 ± 3.80 kg/m^2^) and hyperinsulinemic (37.05 ± 3.88 kg/m^2^) obese subjects (Table 1). Mean lean mass (kg), fat mass (kg), percentual water body constitution (%) and basal metabolic rate (kcal) were significantly higher in both obese subgroups when compared to LS values (Table 1). There were no significant differences between the plasmatic values to triglyceride, cholesterol, HDL-cholesterol total, LDL-cholesterol and very-low density lipoprotein from the LS group compared with OS subgroups (Table 2). Additionally, as can be seen in Figure 2, the basal systolic blood pressure (in mmHg) from HI subjects (130.7 ± 4.71) was significantly higher than from LS (114.8 ± 16.6, P < 0.002) and NI (122.7 ± 12.3, P < 0.024) volunteers while the diastolic blood pressure was different in NI subgroup (84.1 ± 8.4) when compared to LS subjects (73.1 ± 12.4) (P < 0.031).

**Table 2:** The table shows the serum parameter analyses to triglyceride, cholesterol, high-density (HDL), low-density (LDL) and very-low density (VLDL) cholesterol, uric and free fat acids plasma levels from the lean (LS) group, compared with the obese subgroup subjects

	**Control**	**n**	**Norminsulinemic**	**n**	**Hyperinsulinemic**	**n**	**P**	**P**^**1**^	**P**^**2**^	**P**^**3**^
	**(LS)**		**(NI)**		**(HI)**			**LS** ***vs.*** **NI**	**LS** ***vs.*** **HI**	**NI** ***vs.*** **HI**
Cholesterol (mg/dl)	169.33 ± 40.08	12	186.64 ± 27.07	14	184.55 ± 15.97	11	ns	0.045	ns	ns
HDL-cholesterol (mg/dl)	35.08 ± 13.31	13	26.50 ± 4.64	14	26.73 ± 8	11	ns	ns	ns	ns
LDL-cholesterol (mg/dl)	113.17 ± 32.31	12	133.67 ± 27.39	15	139.83 ± 24.56	12	0.040	ns	0.015	ns
VLDL-cholesterol (mg/dl)	18.92 ± 6.99	12	21.87 ± 10.28	15	22.91 ± 8.3	11	ns	ns	ns	ns
Triglyceride (mg/dl)	94.92 ± 34.45	12	111.00 ± 50.13	15	114.91 ± 41.06	11	ns	ns	ns	ns
Uric acid (mg/dl)	4.75 ± 1.53	13	4.49 ± 0.89	15	5.25 ± 1.07	11	ns	ns	ns	0.033
Free fatty acids (mg/dl)	382.05 ± 129.80	12	545.11 ± 454.67	14	396.58 ± 97.1	12	ns	ns	ns	ns

Values are means ± DP; P values for comparison between groups by non-parametric analysis of *Kruskal Wallis*; P, comparing all groups and P^1^, P^2^ and P^3^ comparison between groups.

### Fasting glucose and insulin levels and oral glucose tolerance test (OGTT)

**Figure 3:**
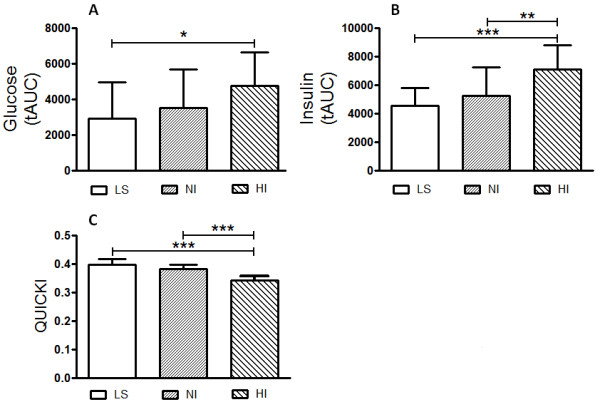
**Graphic representation depicts the total area under curve to glucose (panel A) and insulin (panel B) serum levels after oral glucose test to control lean (LS, n = 12) subjects compared to normoinsulinemic (NI, n = 14) and hyperinsulinemic (HI, n = 12) volunteer subgroups.** Also shows the estimating of quantitative insulin-sensitivity check (QUICKI) index (panel **C**) values for lean and obese subjects. Results are expressed as mean ± SD. The level of significance was set at *P ≤ 0.05 or **P ≤ 0.01 (non-parametric analysis by Kruskal-Wallis test).

The HI subgroup showed higher basal plasmatic insulin and C peptide levels after overnight fasting (Insulin: 10.85 ± 4.09 μg/ml and C peptide: 1.37 ± 0.59 ng/ml), when compared to the LS (Insulin: 4.25 ± 0.86 μg/ml and C peptide: 0.774 ± 0.12 ng/ml, P < 0.0001) and NI (Insulin: 5.51 ± 1.18 μg/ml and C peptide: 1.13 ± 0.82 ng/ml, P < 0.027) groups (Figure 3). The fasting glucose plasma level was significantly different only when obese HI and NI were compared (P < 0.024). Otherwise, the HI group achieved significantly higher plasma glucose concentrations than the LS and NI groups at 30, 60, 90 and 120 minutes, when analyzed the post-absorptive plasma glucose levels after glucose oral loading. Thus, the incremental total area under the curve (tAUC) to glucose (Figure 3A) and insulin (Figure 3B) in HI was significantly higher (P < 0.001) when compared to the LS and NI obese subgroup. The HOMA-IR index, taken as a measure of insulin resistance, was significantly enhanced in HI subjects when compared to the LS and NI subgroups (Figure 1). Reciprocally, the QUICKI index, a measure of insulin sensitivity, was significantly decreased in HI obese subjects when compared with LS and NI volunteers (P < 0.0001) (Figure 3C).

### Cytokines and inflammatory response

**Figure 4:**
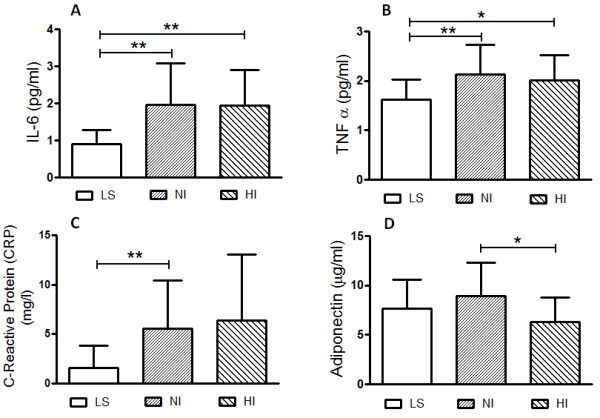
**Graphic representation depicts the circulating plasma concentrations of IL-6 (Panel A), TNF-α (Panel B), and C-reactive protein (Panel C) and Adiponectin (Panel D), measured in basal period to control lean (LS, n = 12) subjects compared to normoinsulinemic (NI, n = 15) and hyperinsulinemic (HI, n = 12) volunteer subgroups.** Results are expressed as mean ± SD. The level of significance was set at *P ≤ 0.05 or ** *P* ≤ 0.01 (non-parametric analysis by Kruskal-Wallis test).

Circulating concentrations of TNF-α, IL-6 and C-reactive protein, measured in basal period, are presented in Table 3. The present study showed a striking inflammatory difference between evaluated lean and obese experimental groups, being significantly greater in obese subjects when compared to the control (Figure 4) subjects. Otherwise, the adiponectin measured in basal period (Table 3; Figure 4D), was significantly enhanced in NI subjects when compared to HI groups (P < 0.04). As presented in Figure 4, the obeses subjects showed an expressive and significant increase of IL-6 (Figure 4A), TNF-α (Figure 4B) and C-reactive protein (Figure 4C) plasma levels compared with the LS group. No difference in leptin plasma level was observed in HI and NI subgroups (Table 3).

**Table 3:** The table shows the circulating plasma concentrations of TNF-α, IL-6 and C-reactive protein, measured in basal period in obese and lean subjects

	**Control**	**n**	**Normoinsulinemic**	**n**	**Hyperinsulinemic**	**n**	**P**	**P**^**1**^	**P**^**2**^	**P**^**3**^
	**(LS)**		**(NI)**		**(HI)**			**LS** ***vs.*** **NI**	**LS** ***vs.*** **HI**	**NI** ***vs.*** **HI**
TNF-α (pg/ml)	1.62 ± 0.40	12	2.13 ± 0.60	15	2.01 ± 0.51	12	0.012	0.005	0.028	ns
IL-6 (pg/ml)	0.90 ± 0.38	12	1.97 ± 1.10	15	1.95 ± 0.96	12	0.001	0.001	0.001	ns
CRP (mg/l)	1.56 ± 2.25	12	5.56 ± 4.89	15	6.37 ± 6.70	12	0.025	0.006	ns	ns
Adiponectin (μg/ml)	7.64 ± 2.94	12	8.91 ± 3.39	15	6.32 ± 2.49	12	ns	ns	ns	0.040
Leptin (μg/ml)	11.99 ± 10.63	12	40.70 ± 21.54	15	33.51 ± 21.82	12	0.006	0.002	0.018	ns

TNF-α - tumor necrosis factor-alpha; IL-6 – interleukin 6; CRP – C reactive protein; Values are means ± DP; P values for comparison between groups by non-parametric analysis of *Kruskal Wallis*; P, comparing all groups: P^1^, P^2^ and P^3^ comparison between groups.

### Renal function data

**Figure 5:**
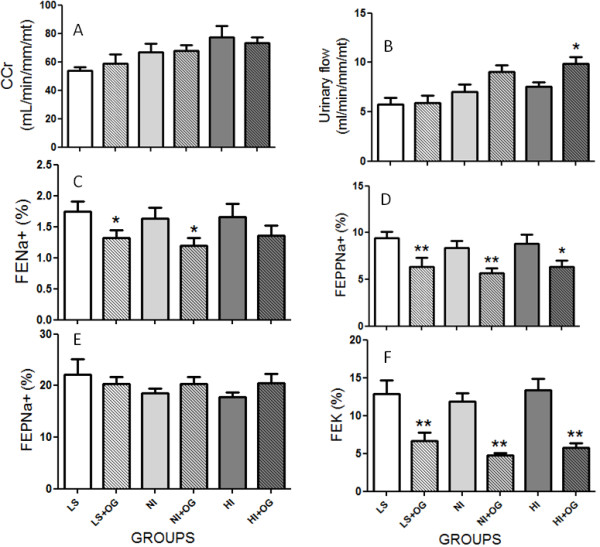
**Bar graph of Creatinine clearance (*C***_**Cr**_**) (Panel A), Urinary flow (Panel B), fractional sodium excretion (FENa) (Panel C), proximal (FEPNa) (Panel E) and post-proximal (FEPPNa) (Panel D) fractional sodium excretion and fractional potassium excretion (FEK) (Panel F) in LS and NI and HI obese subjects before and after oral glucose load (OG).** See Results for statistical analysis details. The data are reported as the means ± SD. The level of significance was set at *P ≤ 0.05 or **P ≤ 0.01 to compare LS *vs.* LS + OG, NI *vs.* NI + OG and HI *vs.* HI + OG subgroups (non-parametric analysis by Kruskal-Wallis test).

The data for renal function of both LS and OS, before and after oral glucose load, are summarized in Figure 5. The urinary flow rates (Figure 5B) did not significantly differ among the groups during the renal tubule sodium handling studies, except after 120 min of glucose load in HI subgroup. The glomerular filtration rate (Figure 5A), estimated by CCr, before and after oral glucose load and, consequently, the calculated glomerular filtered load was unchanged in obese subgroups when compared to age-matched LS subjects. The study shows a significant decrease of the FE_Na_ after oral glucose ingestion in LS (1.74 ± 0.17% *vs.* 1.32 ± 0.12%, P = 0.0318, n = 10) and normoinsulinemic (HI: 1.63 ± 0.18% *vs.* HI + OG: 1.19 ± 0.13%, P = 0.0283, n = 14) but not in hyperinsulinemic obese subgroup (HI: 1.66 ± 0.20% *vs.* 1.36 ± 0.15%, P = 0.1344, n = 12) (Figure 5C). The decreased FE_Na_ was accompanied by significant fall of the FEPP_Na_ (Figure 5D) in LS (LS: 9.41 ± 0.68% *vs.* 6.38 ± 0.92%, P = 0.086), normoinsulinemic (NI: 8.41 ± 0.72% *vs.* 5.66 ± 0.53%, P = 0.0025) and hyperinsulinemic subjects (HI: 8.82 ± 0.98% *vs.* 6.32 ± 0.67%, P = 0.0264) without any change in proximal fractional sodium excretion (FEP_Na_, Figure 5E). This expressive decrease in FEPP_Na_ and FE_Na_ produced by oral glucose intake was followed by significant decrease in kaliuresis (Figure 5F) in the all experimental groups. The experimental groups (LS: 1.24 ± 0.62 or obeses NI: 1.02 ± 0.42 and HI: 1.07 ± 0.35) did not showed significant changes in urinary protein excretion (in mg/dl).

### Interaction analysis

**Figure 6:**
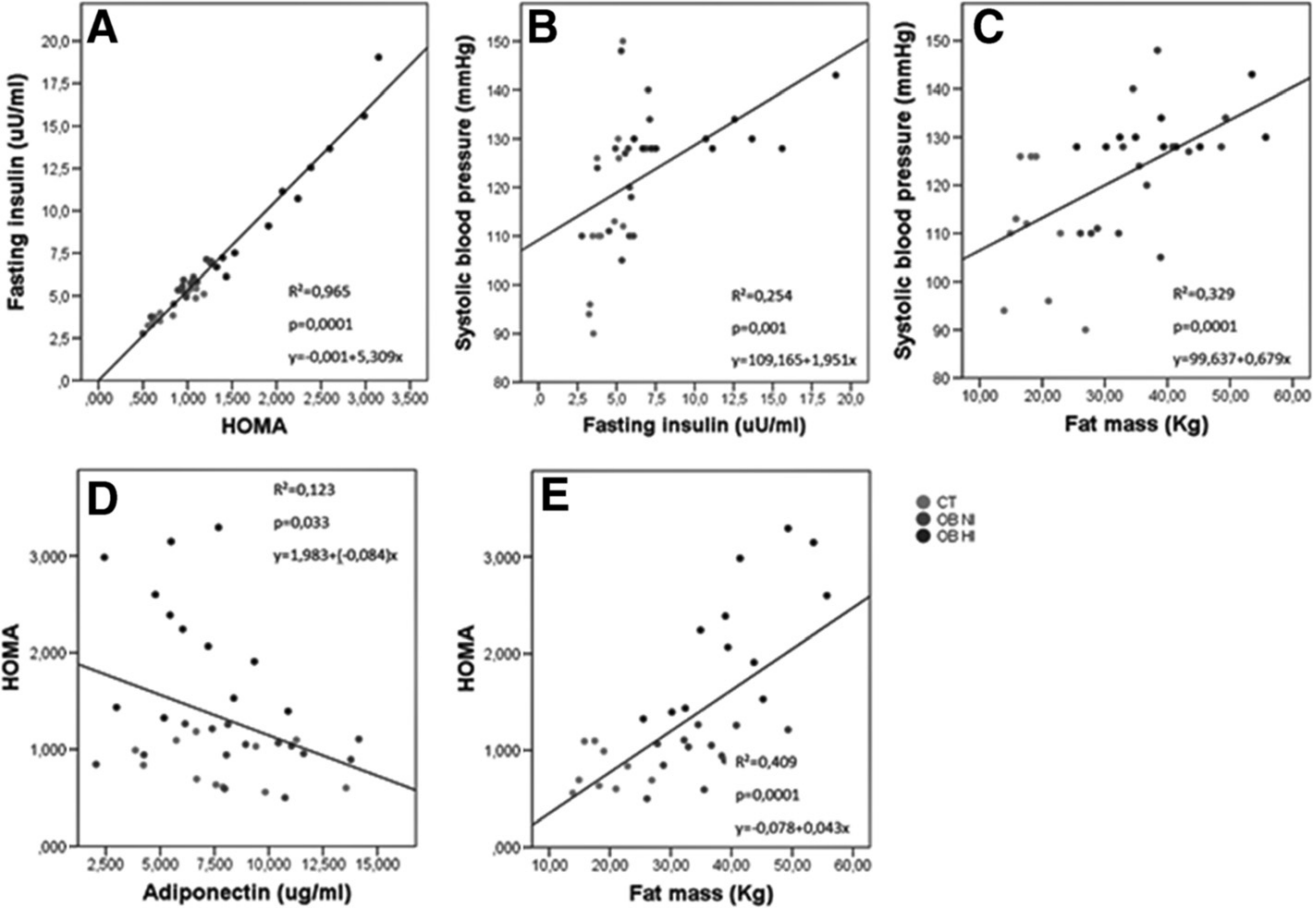
Graphic representation depicts whole lean and obese subject’s data interaction between independent factors related to whole systolic blood pressure (Panel B and C), body constitution (Panel C and E) and estimating HOMA-IR (Panel A, D and E) index values with multiple linear regression analysis.

Figure 6 shows the interaction between independent factors related to systolic blood pressure, body constitution and estimating HOMA-IR index values with multiple linear regression analysis. The data confirms a positive and significant high correlation between basal fasting insulin plasma level, HOMA-IR index, fat body mass and systolic blood pressure (P < 0.001). Despite a high inflammatory activity in both of obese subgroups was not observed any significant correlation between inflammatory markers serum levels and the reduction in all of the studied groups. However, a significant negative Pearson correlation was observed between increased HOMA-IR index and adiponectin plasma levels (R^2^ = 0.123, P < 0.033, n = 39) and between decreased urinary sodium excretion and adiponectin levels (R^2^ = 0.395, P < 0.013, n = 32).

## Discussion

More and more evidence is emerging that highlights the far-reaching consequences of obesity on kidney morphology and function disorders. The prevalence of obesity is increasing by alarming proportions worldwide and more than 20% of the world population is overweight, while nearly 300 million are obese [22-24]. Driving forces for overweight and obesity include increasing sedentary lifestyle and consumption of a western diet high in fat, fructose and salt and their interaction with genetic factors and epigenetic processes [25-27]. This study shows that a hyperinsulinemic (HI: 10.85 ± 4.09 μg/ml) subgroup of well-characterized metabolic syndrome bearers-obese subject but not all obese subjects, show higher glycemic and elevated blood pressure levels when compared to lean (LS) and meanly, normoinsulinemic (NI: 5.51 ± 1.18 μg/ml, P < 0.027) groups. Here, it has been supposed that insulin resistance may result from a cluster of inflammatory and metabolic disorders with an inherent potential for hemodynamic abnormalities particularly, higher blood pressure. Also in the current study, the combination of hyperinsulinemia, higher HOMA-IR (HI: 2.19 ± 0.70 (n = 12) *vs.* LS: 0.83 ± 0.23 (n = 12) and NI: 0.98 ± 0.22 (n = 15), P < 0.0001) associated with lower QUICKI in HI obese when compared with LS and NI volunteers (P < 0.0001), strongly suggests the occurrence of insulin resistance and, as previously demonstrated, a defect in insulin-stimulated peripheral action [28-30]. The link between hyperinsulinemia and higher blood pressure described above does not prove the presence of causal relationships, but experimental findings have shown possible mechanisms, which may account for a putative relationship [31,32]. Otherwise, the prevalence of hypertension in type 2 diabetes mellitus is increased 3-fold, and the coexistence of hypertension in diabetic patients greatly enhances the development of cardiovascular disease and chronic renal failure [31]. The current study shows, although on normal range, a clear and significant increase of the arterial pressure in hyperinsulinemic obeses, characterizing a prehypertension state. Prehypertension is increasingly recognized as a risk factor for cardiovascular disease. This is supported by studies demonstrating the association of increased systolic or diastolic dysfunction in a prehypertension state in genetic or diet-induced rodent models of obesity [8-10]. It is estimated that 37% of the adult population has prehypertension and 40% of these subjects will progress to hypertension within a two-year time frame [8]. By the way, impaired insulin stimulated uptake of glucose associated with impaired vasodilatation has been shown to be early manifestations in insulin resistant models of obesity [11-13,24]. In this respect, increased activation of renin-angiotensin system (RAS) and enhanced oxidative stress within cardiovascular tissue in obesity are important mediators of insulin resistance and vasoconstriction [33]. Although the precise mechanism by which arterial blood pressure enhances in HI obese subjects remain to be elucidated, taking in account our and previous findings we may suppose that systemic inflammatory response, endothelial dysfunction, sympathetic neural and renin-angiotensin overactivity and, renal fluid and electrolyte balance disorder is thought to play a dominant role in the long-term salt and water retention, vascular resistance and consequently, enhanced blood pressure.

In the past two decades, the view of adipose tissue has gone from revolutionary change from inert energy store to the biggest endocrine organ [34]. The current study confirms that in the obese state, the plasma levels of leptin, TNF-α, IL-6 and PCR are increased, and these molecules have been shown to be associated with metabolic inflammation or neologically by metaflammation to review see [35,36] and insulin resistance. Although mechanisms underlying this inflammatory response are not well understood, endoplasmic reticular stress is one of the cellular stress events that activate inflammatory signaling pathways including activation of JNK and mTOR [35,36]. Also, have been shown that TNF-α and IL-6 cause systemic insulin resistance through activation of MAP kinases, protein kinase C (PKC) and SOCS-3 mediated proteasome degradation [24]. In addition to adipose tissue dysfunction, activation of Toll-like receptor 4 (TLR-4) and perhaps other TLRs induced by nutrients excess such as saturated fatty acid, gut derived lipopolysaccharide (LPS), uric acid and/or intestinal dysbiosis contributes significantly to hepatic and systemic inflammatory response in hyperinsulinemic obeses [37,38].

The only hormone displaying an opposite trend is adiponectin. Here, the adiponectin measured in basal period was significantly enhanced in NI subjects when compared to HI groups (P < 0.04). The association between adiponectin and hypertension is evident in clinical studies by showing that hypoadiponectinemia is a risk factor for hypertension independent of insulin resistance and diabetes, while overexpression of adiponectin may also decrease the blood pressure in genetically obese mice [39-41]. Nevertheless, despite the well-established association of adiponectin with metabolic disorders and hypertension, very few studies address the relationship between adiponectin and hypertension at a mechanistic level. Recently, several studies have also focused on the effects of adiponectin on the sympathetic nervous system (SNS), RAS and kidney function. The role of increased SNS activity in insulin resistance and hypertension is increasingly recognized [15,24,31,42]. A disproportionate activation of SNS in the obese state is proposed to play important roles in obesity-associated metabolic dysfunction [43]. Conversely, Tanida et al. [44] have found that adiponectin attenuates blood pressure and sympathetic nerve activity by inhibition of leptin action in the brain. Taking in account our and the above findings, we may speculate that adiponectin, secreted from adipose tissue decreases the risk for insulin resistance, inflammation, and blood pressure elevation in a particular subgroup of normoinsulinemic obeses (Figure 6).

Surprisingly, the current report shows a similar insulin-mediated reduction of post-proximal urinary sodium excretion in lean (LS: 9.41 ± 0.68% *vs.* 6.38 ± 0.92%, P = 0.086) and normoinsulinemic (NI: 8.41 ± 0.72% *vs.* 5.66 ± 0.53%, P = 0.0025) and hyperinsulinemic obese subjects despite of persistent and pronounced insulin plasma levels in HI obese subgroup (HI: 8.82 ± 0.98% *vs.* 6.32 ± 0.67%, P = 0.0264), after oral glucose load, despite elevated insulinemic levels in hyperinsulinemic obeses. This exaggerated enhancement of serum insulin levels in response to an oral glucose load, associated with similar renal sodium reabsorption in NI and HI subjects, separately, could not be enough to explain the high blood pressure development in our specific HI obese subgroup. Thus, the present findings provide additional insights about mechanisms and insulin function during high blood pressure development in obese subjects, beyond of those involved in the renal sodium transport. Furthermore, the equal tubule sodium reabsorption in normoinsulinemic and hyperinsulinemic obeses observed in the current study may argue favorably to decrease renal insulin sensitivity in HI volunteers. The sodium-retaining effect of insulin has been known for a long time [15], but there is no consensus on the exact mechanism of action. Prior studies assume that antinatriuretic effect of insulin, predominantly in the post-proximal renal tubules [15,45], may be due to a fall in filtration fraction, renal sympathetic nerve and/or RAS overstimulation and, suppression of natriuretic peptide release [46]. Also, for all of experimental groups, the contribution of the glucose filtered load to tubule Na^+^ reabsorption via a Na^+^/glucose cotransport or Na^+^/H exchanger should also be considered. Here, the oral glucose load in LS and OS did not change the glomerular filtration rate, estimated by CCr and consequently, the filtered sodium load, suggesting that the antinatriuresis observed was mediated by direct tubular mechanisms. Our data was positively related with an increased post-proximal fractional reabsorption of sodium without enhanced tubular potassium excretion suggesting that insulin action preceded the distal segments of nephron.

Although the renin-angiotensin system (RAS) was not detailed here, the angiotensin II (AngII) effects on sympathetic activation are widely demonstrated by several studies. Under pathophysiological conditions, such as obesity-associated metabolic diseases, the overproduction of AngII plays an important role in the development and progression of insulin resistance, hyperinsulinemia and arterial hypertension. The RAS causes sustained sympathetic overactivity by modulating central neurons in the subfornical organ of the forebrain [47,48]. Interestingly, these neural responses are associated with modulation of peripheral T cell immune responses [47,48] suggesting a link between glucose metabolic disorders and the central regulation of systemic immune and/or inflammatory responses through brain AngII signaling and resultant increased SNS outflow. Thus, we may hypothesize that inappropriate activation of RAS and/or SNS may significantly contribute to this inflammatory response, progression of insulin resistance and kidney disorders in this specific subgroup of hyperinsulinemic obese. We also may not rule out the participation of adiponectin in this process since the plasma adiponectin level is reported to be increased and to show a positive correlation with plasma natriuretic factors in heart disorders (7,14). The current study has shown that the plasma adiponectin level was positively correlated with the enhanced natriuresis in normoinsulinemic obese subjects while it is decreased in HI subgroup. The plasma natriuretic peptide level had the same influence on adiponectin as that of the waist circumference, HDL-cholesterol, and triglycerides. We suppose that positive association between plasma adiponectin and normoinsulinemic individuals in our study that adiponectin may retard the arterial pressure increase and so, may predict a better prognosis or morbidity of cardiovascular disease, specifically on hypertension development, even in obese subjects. The precise mechanism of these phenomena remains unknown.

## Conclusion

In conclusion, this study highlight the importance of dysfunctional inflammatory modulation associated with metabolic disorders (hyperinsulinemia and peripheral insulin resistance), high blood pressure, and renal dysfunction in a particular subgroup of obeses. Supposedly, these metabolic and blood pressure abnormalities are striking attenuate by enhanced adiponectin plasma levels in HI subjects compared to LS and NI subjects. The findings of current study may also state that a maladaptive inflammatory response seems to be central to obese-associated cardiovascular disorder and insulin resistance. Prospective investigations are therefore warranted to determine whether high adiponectin levels are related to better prognosis or future morbidity of metabolic or kidney disorders even in obese subjects.

## Abbreviations

OGTT: Oral glucose tolerance test; BMI: Body mass index; HOMA-IR: Homeostasis model assessment of insulin resistance; QUICKI index: Quantitative insulin-sensitivity check index, HI, Hyperinsulinemia; NI: Normoinsulinemic, LS, Lean subjects; OS: Obese subjects; HDL-cholesterol: High-density; LDL-cholesterol: Low-density cholesterol; VLDL-cholesterol: Very low-density cholesterol; CCr: Creatinine clearance, CLi, Lithium clearance, FE_Na,_ Fractional sodium excretion; FE_K_: Fractional potassium excretion; FEP_Na_: Fractional proximal sodium excretion; FEPP_Na_: Fractional post-proximal sodium excretion; RAS: Renin-angiotensin system; AngII: Angiotensin II; tAUC: Total area under curve; TNF-α: Tumor necrosis alpha factor; IL-6: Interleukin 6; CRP: C-reactive protein, SNS, Sympathetic nervous system; MAP kinases: Mitogen-activated protein kinases; PKC: Protein kinase C; SOCS-3: Suppressor of cytokine signaling 3; JNK: c-Jun N-terminal kinases; mTOR: Mammalian target of rapamicina; TLR-4: Toll-like receptor 4 and lipopolysaccharide (LPS).

## Competing interest

The authors declare that they have no competing interests.

## Authors’ contributions

ARA carried out the experimental studies, participated in the volunteer’s selection, renal test, biochemical analysis and drafted the manuscript. MBZ, ALS and ME carried out the bioelectrical impedance and energy expenditure studies and biochemical analysis. ARA and JAG participated in the design of the study and performed the statistical analysis. JAG and SMA conceived of the study, and participated in its design and coordination and helped to draft the manuscript. All authors read and approved the final manuscript.

## References

[B1] Olshansky SJ Passaro DJ Hershow RC Layden J Carnes BA Brody J Hayflick L Butler RN Allison DB Ludwig DS A potential decline in life expectancy in the United States in the 21st century N Engl J Med 2005 352 1138 1145 10.1056/NEJMsr043743 15784668

[B2] Pereira-Lancha LO Campos-Ferraz PL Lancha AH Jr Obesity: considerations about etiology, metabolism, and the use of experimental models Diabetes Metab Syndr Obes 2012 5 75 87 22570558 10.2147/DMSO.S25026 PMC3346207

[B3] Parker DR Weiss ST Troisi R Cassano PA Vokonas PS Lansberg L Relationship of dietary saturated fatty acids and body habitus to serum insulin concentrations: the Normative Aging Study Am J Clin Nutr 1993 58 129 136 8338037 10.1093/ajcn/58.2.129

[B4] Haslam DW James WP Obesity Lancet 2005 366 1197 1209 10.1016/S0140-6736(05)67483-1 16198769

[B5] Pinhal CS Lopes A Torres DB Felisbino SL Rocha Gontijo JA Boer PA Time-course morphological and functional disorders of the kidney induced by long-term high-fat diet intake in female rats Nephrol Dial Transplant 2013 28 10 2464 2476 10.1093/ndt/gft304 24078639

[B6] Grassi G Sympathetic overdrive and cardiovascular risk in the metabolic syndrome Hypertens Res 2006 29 839 847 10.1291/hypres.29.839 17345783

[B7] Aroor AR McKarns S DeMarco FG Jia G Sowers JR Maladaptive immune and inflammatory pathways lead to cardiovascular insulin resistance Metabolism 2013 http://dx.doi.org/10.1016/j.metabol.2013.07.001 10.1016/j.metabol.2013.07.001 PMC3809332 23932846

[B8] Faselis C Doumas M Kokkinos JP Panagiotakos D Kheirbek R Sheriff HM Hare K Papademetriou V Fletcher R Kokkinos P Exercise capacity and progression from prehypertension to hypertension Hypertension 2012 60 333 338 10.1161/HYPERTENSIONAHA.112.196493 22753224

[B9] DeMarco VG Johnson MS Ma L Pulakat L Mugerfeld I Hayden MR Garro M Knight W Britton SL Koch LG Sowers JR Overweight female rats selectively breed for low aerobic capacity exhibit increased myocardial fibrosis and diastolic dysfunction Am J Physiol Heart Circ Physiol 2012 302 H1667 H1682 10.1152/ajpheart.01027.2011 22345570 PMC3330799

[B10] DeMarco VG Ford DA Henriksen EJ Aroor AR Johnson MS Habibi J Ma L Yang M Albert CJ Lally JW Ford CA Prasannarong M Hayden MR Whaley-Connell AT Sowers JR Obesity-related alterations in cardiac lipid profile and nondipping blood pressure pattern during transition to diastolic dysfunction in male db/db mice Endocrinology 2013 154 159 171 10.1210/en.2012-1835 23142808 PMC3529378

[B11] Hall JE The kidney, hypertension, and obesity Hypertension 2003 41 625 633 10.1161/01.HYP.0000052314.95497.78 12623970

[B12] Abel ED O'Shea KM Ramasamy R Insulin resistance: metabolic mechanisms and consequences in the heart Arterioscler Thromb Vasc Biol 2012 32 2068 2076 10.1161/ATVBAHA.111.241984 22895668 PMC3646067

[B13] Wong C Marwick TH Obesity cardiomyopathy: pathogenesis and pathophysiology Nat Clin Pract Cardiovasc Med 2007 4 436 443 10.1038/ncpcardio0943 17653116

[B14] Wang ZV Scherer PE Adiponectin, cardiovascular function, and hypertension Hypertension 2008 51 8 14 10.1161/HYPERTENSIONAHA.107.099424 17998473

[B15] Gontijo JA Muscelli EO Reduced renal sodium excretion in primary hypertensive patients after an oral glucose load Braz J Med Biol Res 1996 29 10 1291 1299 9181099

[B16] Souza ML Berardi EO Gontijo JA Leme Júnior CA Cavicchio JR Saad MJ Insulin resistance and myocardial hypertrophy in the attenuated reduction in mean arterial pressure after a glucose load in hypertensive patients Braz J Med Biol Res 1995 28 9 967 972 8580884

[B17] Tanaka RC Gontijo JA Urinary acidification and renal sodium handling in a case of renal Fanconi syndrome Nephron 1998 78 3 339 340 10.1159/000044949 9546700

[B18] Matthews DR Hosker JP Rudenski AS Naylor BA Treacher DF Turner RC Homeostasis model assessment: insulin resistance and beta-cell function from fasting plasma glucose and insulin concentrations in man Diabetologia 1985 28 7 412 419 10.1007/BF00280883 3899825

[B19] Katz A Nambi SS Mather K Baron AD Follmann DA Sullivan G Quon MJ Quantitative insulin sensitivity check index: a simple, accurate method for assessing insulin sensitivity in humans J Clin Endocrinol Metab 2000 85 7 2402 2410 10.1210/jcem.85.7.6661 10902785

[B20] Furlan FC Marshall PS Macedo RF Carvalheira JB Michelotto JB Gontijo JA Acute intracerebroventricular insulin microinjection after nitric oxide synthase inhibition of renal sodium handling in rats Life Sci 2003 72 2561 2569 10.1016/S0024-3205(03)00170-X 12672502

[B21] Mesquita FF Gontijo JA Boer PA Maternal undernutrition and the offspring kidney: from fetal to adult life Braz J Med Biol Res 2010 43 11 1010 1018 10.1590/S0100-879X2010007500113 21049242

[B22] Reaven GM Insulin resistance: the link between obesity and cardiovascular disease Med Clin North Am 2011 95 875 892 10.1016/j.mcna.2011.06.002 21855697

[B23] McCullough AJ Epidemiology of the metabolic syndrome in the USA J Dig Dis 2011 12 333 340 10.1111/j.1751-2980.2010.00469.x 21091931

[B24] Aroor AR Mandavia CH Sowers JR Insulin resistance and heart failure: molecular mechanisms Heart Fail Clin 2012 8 609 617 10.1016/j.hfc.2012.06.005 22999243 PMC3457065

[B25] Stanhope KL Role of fructose-containing sugars in the epidemics of obesity and metabolic syndrome Annu Rev Med 2012 63 329 343 10.1146/annurev-med-042010-113026 22034869

[B26] Garver WS Newman SB Gonzales-Pacheco DM Castillo JJ Jelinek D Heidenreich RA Orlando RA The genetics of childhood obesity and interaction with dietary macronutrients Genes Nutr 2013 8 271 287 10.1007/s12263-013-0339-5 23471855 PMC3639324

[B27] Drong AW Lindgren CM McCarthy MI The genetic and epigenetic basis of type 2 diabetes and obesity Clin Pharmacol Ther 2012 92 707 715 10.1038/clpt.2012.149 23047653 PMC7116747

[B28] Ferrannini E Buzzicoli G Bonadonna RN Insulin resistance in essential hypertension Eng J Med 1985 317 350 357 10.1056/NEJM198708063170605 3299096

[B29] Florey CV Uppal S Lowy C Relation between blood pressure, weight, and plasma sugar serum insulin levels in schoolchildren age 9–12 years in Westland Holland 1976 1 1368 1371 10.1136/bmj.1.6022.1368 PMC1640120 1276694

[B30] Minicardi V Camellini L Belloidi G Ferrannini E Evidence for an association of high blood pressure and hyperinsulinemia in obese man J Clin Endocrinol Metabol 1986 62 1302 1304 10.1210/jcem-62-6-1302 3517032

[B31] Sowers JR Diabetes mellitus and vascular disease Hypertension 2013 61 943 947 10.1161/HYPERTENSIONAHA.111.00612 23595139 PMC3648858

[B32] Witteles RM Fowler MB Insulin-resistant cardiomyopathy clinical evidence, mechanisms, and treatment options J Am Coll Cardiol 2008 51 93 102 10.1016/j.jacc.2007.10.021 18191731

[B33] Whaley-Connell A Sowers JR Oxidative stress in the cardiorenal metabolic syndrome Curr Hypertens Rep 2012 14 360 365 10.1007/s11906-012-0279-2 22581415 PMC3636553

[B34] Scherer PE Adipose tissue: from lipid storage compartment to endocrine organ Diabetes 2006 55 1537 1545 10.2337/db06-0263 16731815

[B35] Kalupahana NS Moustaid-Moussa N Claycombe KJ Immunity as a link between obesity and insulin resistance Mol Aspects Med 2012 33 26 34 10.1016/j.mam.2011.10.011 22040698

[B36] Gregor MF Hotamisligil GS Inflammatory mechanisms in obesity Annu Rev Immunol 2011 29 415 445 10.1146/annurev-immunol-031210-101322 21219177

[B37] Shen J Obin MS Zhao L The gut microbiota, obesity and insulin resistance Mol Aspects Med 2013 34 39 58 10.1016/j.mam.2012.11.001 23159341

[B38] Brown K DeCoffe D Molcan E Diet-induced dysbiosis of the intestinal microbiota and the effects on immunity and disease Nutrients 2012 4 1095 1119 10.3390/nu4081095 23016134 PMC3448089

[B39] Ohashi K Kihara S Ouchi N Kumada M Fujita K Hiuge A Hibuse T Ryo M Nishizawa H Maeda N Maeda K Shibata R Walsh K Funahashi T Shimomura I Adiponectin replenishment ameliorates obesity-related hypertension Hypertension 2006 47 1108 1116 10.1161/01.HYP.0000222368.43759.a1 16651465

[B40] Iwashima Y Katsuya T Ishikawa K Ouchi N Ohishi M Sugimoto K Fu Y Motone M Yamamoto K Matsuo A Ohashi K Kihara S Funahashi T Rakugi H Matsuzawa Y Ogihara T Hypoadiponectinemia is an independent risk factor for hypertension Hypertension 2004 43 1318 1323 10.1161/01.HYP.0000129281.03801.4b 15123570

[B41] Chow WS Cheung BM Tso AW Xu A Wat NM Fong CH Ong LH Tam S Tan KC Janus ED Lam TH Lam KS Hypoadiponectinemia as a predictor for the development of hypertension: a 5-year prospective study Hypertension 2007 49 1455 1461 10.1161/HYPERTENSIONAHA.107.086835 17452504

[B42] Gontijo JA Garcia WE Figueiredo JF Silva-Netto CR Furtado MR Renal sodium handling after noradrenergic stimulation of the lateral hypothalamic area in rats Braz J Med Biol Res 1992 25 937 942 1342841

[B43] Mancia G Bousquet P Elghozi JL Esler M Grassi G Julius S Reid J Van Zwieten PA The sympathetic nervous system and the metabolic syndrome J Hypertens 2007 25 909 920 10.1097/HJH.0b013e328048d004 17414649

[B44] Tanida M Shen J Horii Y Matsuda M Kihara S Funahashi T Shimomura I Sawai H Fukuda Y Matsuzawa Y Nagai K Effects of adiponectin on the renal sympathetic nerve activity and blood pressure in rats Exp Biol Med (Maywood) 2007 232 390 397 17327472

[B45] Kirchner KA Insulin increases loop segment chloride reabsorption in the euglycemic rat Am J Physiol 1988 255 F1206 F1213 3059825 10.1152/ajprenal.1988.255.6.F1206

[B46] Anderson EA Hoffman RP Balon TW Sinkey CA Mark AL Hyperinsulinemia produces both sympathetic neural activation and vasodilation in normal human JCI 1991 87 2246 2252 10.1172/JCI115260 2040704 PMC296986

[B47] Abboud FM Harwani SC Chapleau MW Autonomic neural regulation of the immune system: implications for hypertension and cardiovascular disease Hypertension 2012 59 755 762 10.1161/HYPERTENSIONAHA.111.186833 22331383 PMC3313828

[B48] Harrison DG Marvar PJ Titze JM Vascular inflammatory cells in hypertension Front Physiol 2012 3 128 22586409 10.3389/fphys.2012.00128 PMC3345946

